# Role of the mechanical microenvironment on CD-44 expression of breast adenocarcinoma in response to radiotherapy

**DOI:** 10.1038/s41598-023-50473-x

**Published:** 2024-01-03

**Authors:** Crescenzo Frascogna, Rocco Mottareale, Giuseppe La Verde, Cecilia Arrichiello, Paolo Muto, Paolo A. Netti, Mariagabriella Pugliese, Valeria Panzetta

**Affiliations:** 1 Center for Advanced Biomaterials for Healthcare @CRIB, Italian Institute of Technology, Largo Barsanti e Matteucci 53, 80125 Naples, Italy; 2 Department of Chemical, Materials and Production Engineering, University of Naples Federico II, Piazzale Vincenzo Tecchio, 80125 Naples, Italy; 3 Department of Physics “E. Pancini”, University of Naples Federico II, Via Cinthia, 80126 Naples, Italy; 4 Istituto Nazionale di Fisica Nucleare, INFN Sezione di Napoli, Via Cinthia Ed. 6, 80126 Naples, Italy; 5 Radiotherapy Unit, Istituto Nazionale Tumori-IRCCS-Fondazione “G. Pascale”, Via Semmola, 53, 80131 Naples, Italy; 6 Interdisciplinary Research Centre On Biomaterials CRIB, University of Naples Federico II, Piazzale Vincenzo Tecchio, 80125 Naples, Italy

**Keywords:** Biomedical engineering, Biophysics

## Abstract

The biological effects of ionizing radiation are exploited in the clinical practice of radiotherapy to destroy tumour cells while sparing the surrounding normal tissue. While most of the radiotherapy research focused on DNA damage and repair, recently a great attention is going to cells' interactions with the mechanical microenvironment of both malignant and healthy tissues after exposure. In fact, the stiffness of the extracellular matrix can modify cells' motility and spreading through the modulation of transmembrane proteins and surface receptors' expression, such as CD-44. CD-44 receptor has held much interest also in targeted-therapy due to its affinity with hyaluronic acid, which can be used to functionalize biodegradable nanoparticles loaded with chemotherapy drugs for targeted therapy. We evaluated changes in CD-44 expression in two mammary carcinoma cell lines (MCF10A and MDA-MB-231) after exposure to X-ray (2 or 10 Gy). To explore the role of the mechanical microenvironment, we mimicked tissues' stiffness with polyacrylamide's substrates producing two different elastic modulus values (0.5 and 15 kPa). We measured a dose dependent increase in CD-44 relative expression in tumour cells cultured in a stiffer microenvironment. These findings highlight a crucial connection between the mechanical properties of the cell's surroundings and the post-radiotherapy expression of surface receptors.

## Introduction

The radiobiological effects associated with cellular exposure to ionizing radiation (IR) are known and recognized in clinical practice for the treatment of tumours in radiotherapy (RT). RT can induce dramatic consequences for cells, producing both direct and indirect injuries to DNA, resulting in the formation of lethal chromosome aberrations (double-strand breaks described by the Linear-Quadratic model^1,2^) ultimately leading to cell death or inhibited growth^3–5^. The prevailing consensus now underscores not only the central role of genomic DNA integrity but also emphasizes the significance of the cytoskeleton (CSK) and extracellular matrix (ECM) in various biological processes. The perturbation of these structures emerges as a critical determinant in cancer progression. Empirical evidence has specifically demonstrated that neoplastic cells exhibit a cytoskeletal architecture characterized by diminished organization and structural coherence. Consequently, these cells display reduced mechanical robustness and decreased cyto-adhesive properties, presenting a stark contrast to their non-neoplastic counterparts^6–10^. Noteworthy is the fact that alterations in the structure of the CSK exert a significant impact on the motility and heightened metastatic propensity of cancer cells. Additionally, changes in the composition and stiffness of the surrounding tissue (i.e., ECM) can induce cells’ growth and activate processes that lead to spreading and invasion^11–14^. In the current context, the research landscape has extensively explored the RT impact on cell behavior, neglecting that produced on ECM content and structural organization^11,15–18^. However, a noteworthy shift is necessary, directing attention towards a more comprehensive analysis of IR on cancer mechanobiology. This evolving focus not only delves into the study of radio-induced DNA damage, a critical element affecting the integrity and survival of cancer cells but also extends to investigate the role of IR in altering the physical interactions of cells within their surrounding environment^11–13^.

These alterations are closely tied to radiation dose, post-irradiation time, and ECM mechanical rigidity. This shift in perspective finds support in in vitro experiments, where the effects of radiation on cell motility were found to be contingent on the cell phenotype and the delivered radiation dose^19–21^. Our group's observations further contribute to this understanding, noting a significant increase in the mechanical properties of tumor fibroblasts, promoting cell adhesion and reducing migration, following irradiation with 250 kV and 6 MV^22–24^. These findings also underscore the active role of ECM mechanics in mediating cellular responses to radiation, as further evidenced in the context of breast cancer. The aggressive breast adenocarcinoma MDA-MB-231 cell line displays significant alterations in biophysical characteristics such as cell area and nuclear morphology when cultured on a substrate mimicking the mechanical rigidity of tumor microenvironments^25^. Notably, the effects were less pronounced when cells engaged with a substrate simulating physiological conditions. Moreover, an interesting phenomenon emerged in terms of cell migratory behavior. Both MDA-MB-231 and the healthy epithelial MCF10A cell line exhibited a notable decrease in migration velocity on the softer substrate, that may be attributed to a potential radioprotective role associated with physiological ECM. This radioprotective influence appears to limit cell motility and suppress invasive tendencies, as evident by the reduction of migration speed^22–24^. In this context, numerous studies have presented conflicting evidence regarding the impact of IR on cell motility. In fact, IR can induce both increase and decrease of cell velocity and directionality depending on specific cell types and experimental conditions^13,26–29^. In the clinical scenario, certain trials suggest a potential association between radiation and the increased risk of metastasis. This risk may stem from factors such as the radio-induced release of tumor cells into the cardiovascular system, systemic impacts on both tumor and normal tissue due to irradiation, and phenotypic alterations induced by radiation^30,31^.

Hence, research efforts have been directed towards the exploration of novel integrated strategies involving RT and chemotherapy. These strategies aim at mitigating the adverse radio-induced effects, particularly in the context of post-exposure metastasis formation. This approach leverages the active targeting capabilities of chemotherapy drugs via proteins and receptors that are overexpressed in cancer cells due to radiation's impact on the CSK and ECM structures. In this context, considerable attention has been directed towards CD-44, a transmembrane receptor, also recognized as H-CAM (*Hermes Ag and human phagocytic glycoprotein-1*). Comprising a diverse family of transmembrane glycoproteins with 20 distinct isoforms, CD-44 is shaped through post-transcriptional modifications and a high rate of alternative splicing^32,33^. It actively participates in various adhesion processes with ECM components and facilitates the transduction of intercellular signals, playing a pivotal role in cell adhesion and mobility^34,35^. Its verified involvement in the progression of tumors and the metastatic process makes CD-44 a subject of substantial investigation^36–40^. CD-44 has been found as a surface marker of Cancer Stem Cells (CSCs), a distinctive subpopulation of cancer cells implicated in tumour initiation, invasion, recurrence, and resistance to chemo-radiotherapy^41^: in this context, CD-44 correlates with local tumour control after RT of early laryngeal cancer, lung adenocarcinoma, oropharyngeal squamous cell carcinoma and pancreatic cancer as its expression bears the potential to predict the RT outcome by assessment of CSCs density^42–48^.

**Figure 1:**
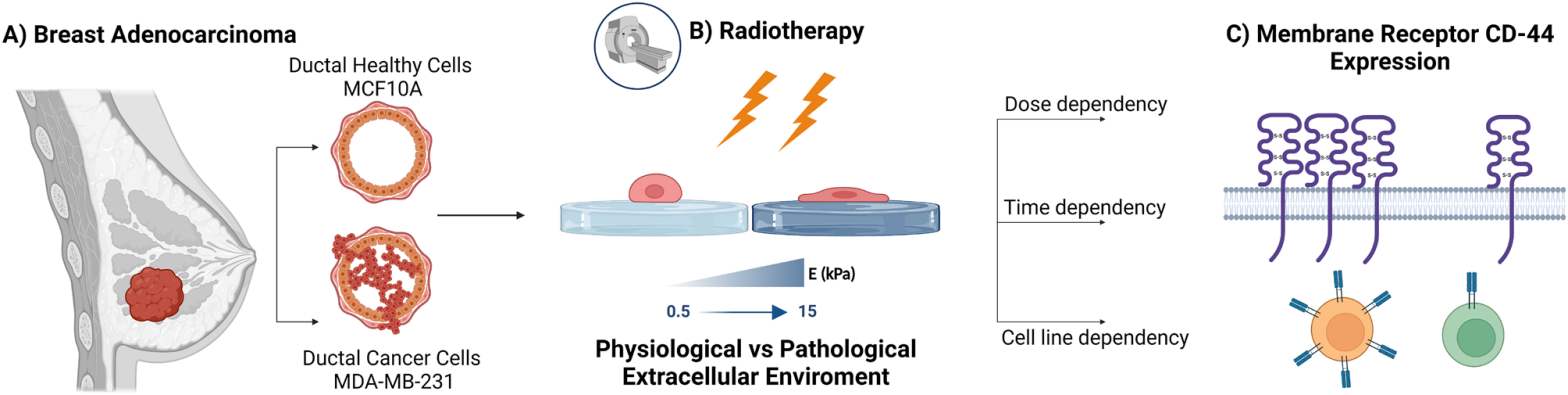
Two cell lines, one healthy (normal mammary epithelial, MCF10A, **A**) and the other tumour (aggressive breast adenocarcinoma, triple negative, MDA-MB-231, **A**), on two polyacrylamide (PAAm) substrates with stiffnesses across a range of pathophysiological range of values (0.5–15 kPa, **B**). The expression of CD-44 was evaluated through immunofluorescence 24 and 72 h after the exposure to two different doses of X-rays (2 and 10 Gy, **C**).

The evidence of a CSC-related radio-resistance in breast cancer was observed by Philips et al.^49^: CD24^−/low^/CD-44^+^ cancer-initiating cells were isolated from MCF-7 and MDA-MB-231 breast cancer monolayer cultures and propagated as spheroids in mammospheres. Fractionated radiation appeared to increase the percentage of CD24^−/low^/CD-44^+^ cells suggesting that the relative radio-resistance of this subset may lead to their expansion during a course of RT^50^. Nevertheless CSC-related chemo-radio-resistance in breast cancer has been further investigated^50,51^, the existence of an intrinsic increase in CD-44 expression due to RT has not been already quantified. Our work’s aim was to investigate the role of radiation on CD-44 expression on both normal and tumour cells for different mechanical microenvironmental conditions since a possible relative increase in expression would open to the definition of combined radio-chemotherapy strategies for breast adenocarcinoma treatment. The heightened expression of CD-44 in cancer cell lines, including MCF-7, MDA-MB-468, and MDA-MB-231^52^, along with its strong affinity for hyaluronic acid (HA), has garnered significant attention in the context of breast cancer treatment. Breast cancer, being one of the most prevalent and resilient neoplasms, has been extensively studied, with the gold standard treatment often involving combined radio-pharmacological therapy^53^. Notably, HA can be utilized for the functionalization of biocompatible and biodegradable nanoparticles carrying chemotherapy drugs^54–56^. This strategy seeks selective uptake by breast cancer cells, achieved through active targeting facilitated by the interaction between HA and the CD-44 receptor. Here, we evaluated CD-44 expression after irradiation for different dose conditions and post-irradiation times, as part of a conventional RT plan (Fig. 1).

The analysis was performed on two cell lines, the normal mammary epithelial, MCF10A, and the aggressive breast adenocarcinoma, triple negative, MDA-MB-231. Immunofluorescence analysis was conducted to assess CD-44 expression at the single-cell level, allowing determination of the transmembrane protein concentration relative to cell surface area. CD-44 expression was examined at two different post-exposure times (24 and 72 h) with cells cultured on polyacrylamide (PAAm) substrates, varying in stiffness (Young's modulus of 0.5 and 15 kPa). This design not only considered the time-dose factor but also evaluated the influence of the mechanical microenvironment on cellular expression properties. These findings suggest potential avenues for future investigations in optimizing combined radio-targeted-nanomedicine strategies for eradicating tumor cell populations.

## Materials and methods

### Preparation of substrate and mechanical characterization

Polyacrylamide substrates were prepared following a previously published method^57^, with some minor adjustments. Here is a concise description of the process: Glass-bottom culture dishes (World Precision Instruments, FD35-100) were surface-treated with 3-aminopropyltriethoxysilane (Sigma-Aldrich, St. Louis, MO, USA) for 20 min. Afterward, they were extensively washed with water to remove excess reagent. A Polyacrylamide mixture was created using 40% acrylamide and 2% methylene-bis-acrylamide in a phosphate-buffered saline (PBS) solution. Two different final concentrations were prepared: 2.5% acrylamide/0.15% methylene-bis-acrylamide and 8% acrylamide/0.1% methylene-bis-acrylamide. These concentrations correspond to stiffness levels of 0.5 kPa and 15 kPa (Young's modulus), respectively. Polymerization was initiated by adding 1/100th of the total volume of 10% ammonium persulfate and 1/1000th of the total volume of N,N,N′,N′-tetramethylethylenediamide (TEMED, Sigma-Aldrich, St. Louis, MO, USA). To create the polyacrylamide substrates, 15 μl of the acrylamide/methylenebis-acrylamide mixture was pipetted onto the treated glass-bottom culture dishes. The mixture was then covered with a 20-mm coverslip. Then, after 20 min, the coverslip was removed, and PBS was added to the dish. The substrates were soaked with a penicillin–streptomycin solution overnight and then exposed to UV light emitted by a germicidal lamp for 1 h. After sterilizations, substrates were functionalized with collagen by using a bifunctional photoreactive crosslinker (sulfosuccinimidyl 6-(4′-azido-2′-nitrophenylamino) hexanoate, sulfo-SANPAH (Fischer Scientific, Loughborough, UK). The sulfo-SANPAH solution was diluted in water (double-distilled) at a final concentration of 0.2 mg/mL, placed on PAAm substrates, and exposed to 365 nm UV for 10 min. After washing with PBS, the substrates were incubated with a solution of bovine type I collagen (Sigma-Aldrich, C4243, St. Louis, MO, USA) at the final concentration of 50 μg/mL in bi-distilled water for 1 h at 37 °C. Finally, samples were washed with PBS.

### Cell culture

MDA-MB-231 cells (nicely donated by Francesco Paolo Cammarata, Institute of Molecular Bioimaging and Physiology, National Research Council IBFM-CNR, 90015 Cefalù, Italy) were cultured in Dulbecco’s Modified Eagle′s Medium/Nutrient Mixture F-12 Ham (Sigma, St. Louis, MO, USA) supplemented with 10% foetal bovine serum (FBS, Gibco, Eggenstein, Germany), 1% l-glutamine (Sigma, St. Louis, MO, USA) and 1% penicillin–streptomycin (Sigma, St. Louis, MO, USA).

MCF10A cells (nicely donated by Stefano Piccolo, AIRC Institute of Molecular Oncology, 20139 Milan, Italy) was cultured in the same basal medium but supplemented with 5% horse serum (HS), 1% l-glutamine (Sigma, St. Louis, MO, USA), 1% penicillin–streptomycin (Sigma, St. Louis, MO, USA), 0.1% epithelial growth factor (EGF), 0.1% insulin and 0.1% hydrocortisone.

### Cell irradiation

MCF10A and MDA-MB-231 cell lines underwent exposure to X-rays, delivered via the LINAC Synergy Agility system (ELEKTA), utilizing a 6 MV energy beam. To administer radiation doses of 2 and 10 Gy, we employed 3D conformal radiation therapy (3D-CRT) treatment plans generated using the Monaco v5.11.03 treatment planning station by Elekta, as previously implemented^58^. For the experimental setup, cells were positioned in a Petri dish between two solid water phantom slabs (ScandiDos Delta-4 Calibration Phantom), each measuring 3 and 5 cm, and were irradiated from opposing fields (180° gantry rotation). A dose rate of 200 UM/min was selected, and the prescribed doses were delivered on a uniform square field measuring 20 × 20 cm^2^ at the cell level. The choice of 2 Gy represents the conventional fractional dose administered in standard treatments, whereas 10 Gy corresponds to the dose used in specific clinical practices, typically as a post-operative boost treatment. Following irradiation, the cells were incubated at 37 °C and 5% CO_2_. Subsequently, cells were fixed 24 h and 72 h post-irradiation using a 4% paraformaldehyde solution in 1× PBS for 20 min at room temperature.

### Immunostaining

MCF10A and MDA-MB-231 cells, fixed at 24 h and 72 h post irradiation, were immunostained for the analysis of cells’ spreading and CD-44 receptor’s expression.

Cells’ spreading was investigated for the different experimental conditions using the following protocol for the immunofluorescence: cell membrane was stained for 30 min with WGA (Wheat Germ Agglutinin, W11261 Thermo Fisher Scientific) conjugated with the fluorescent probe Rhodamine (λ_excitation_ ~ 550 nm, λ_emission_ ~ 570–590 nm) at 1/200 dilution in HBSS buffer (Hanks’ Balanced Salt Solution, X0507 Microgem); cell nuclei were stained in Hoechst 33342 (Sigma-Aldrich St. Louis, MO, USA) (λ_excitation_ ~ 360 nm, λ_emission_ ~ 460–470 nm) at 1/1000 dilution in PBS.

CD-44 expression was investigated using the following protocol for the immunofluorescence: cell membrane was stained for 30 min with Cell Tracker™ (C34565 Thermo Fisher Scientific) Red CMTPX (λ_excitation_ ~ 577 nm, λ_emission_ ~ 602 nm) at 1/1000 dilution in PBS. Cells were then permeabilized with 0.1% Triton X100 (Sigma-Aldrich, T9284, St. Louis, MO, USA in 1× PBS for 15 min and blocked with 3% bovine serum albumin (BSA, A9418 Sigma-Aldrich St. Louis, MO, USA) at 0.1% dilution in Triton X100 in 1× PBS for 1 h. Cells were incubated with Mouse CD-44 antibody (AB6124 Abcam) at 1/400 dilution in 3% BSA- 0.1% Triton X100 in 1× PBS for 1.5 h; then cells were incubated with Alexa Fluor 488 (λ_excitation_ ~ 488 nm, λ_emission_ ~ 520 nm) conjugated goat anti-mouse secondary antibody at 1/200 dilution in 3% BSA-Triton X100 in 1× PBS for 1.5 h.

### Cell adhesion analysis

Images of cells were acquired using a Zeiss LMS-800 confocal microscope and a 10× objective. Images were imported into ImageJ software (NIH, Bethesda, MD, USA) for the quantification of cell spreading area in the different experimental conditions. Individual cells were thresholded manually based on WGA-Rhodamine and Hoechst signals for cells’ membranes and nuclei staining respectively, and their spreading areas were determined from cellular membranes’ signals using the “Measure” command in ImageJ.

### CD-44 expression analysis

In this scientific study, images of individual cells were captured under various experimental conditions using a Zeiss LMS-800 confocal microscope with a 63× objective. These images were then processed using ImageJ software to quantify the expression of the CD-44 receptor in each cell. The process involved projecting the CD-44 signal onto the cellular adhesion plane, defining the region of interest (ROI) through the cell membrane signal, and quantifying CD-44's intensity as "Integrated Density" using ImageJ tools. Therefore, background removal was performed to craft a reliable rendering of the cell's architecture with Imaris 3D software.

Imaris was used to reconstruct a 3D model of the single cell, known as "Surface," through a series of steps involving pre-processing, segmentation, and labelling. Imaris Surface models allowed the identification and measurement of various structures, including area, volume, intensity, position, and elliptical features. The reconstruction process in Imaris involved an octal tree structure, and the resolution level for cell membrane reconstruction was set to 0.3 μm. The fluorescence signal's intensity level could be adjusted by modifying the threshold. The resulting 3D model was used to quantify the total area of the cell membrane (µm^2^), and the fluorescence intensity data for CD-44 (i.e., "Integrated Density") were normalized to account for variations in cell morphology across samples. This normalization ensured that the expression results were independent of the cell's specific morphological characteristics.

### Statistical analysis

All results were reported for the different experimental conditions considered in the form of boxplots used for the graphical representation of the distribution of individual samples, through statistical indices of dispersion and position*:* each box is delimited by the first and third quartile and divided within it by the median; the segments of each box identify the minimum and the maximum values respectively. The normality of data was checked by the Shapiro–Wilk test (p values < 0.05 indicates non-normal distribution). Statistical comparisons were performed with a Student’s unpaired test when data exhibit a normal distribution. Otherwise, a nonparametric Kruskal–Wallis test was used (Tables S1, S2, S3). Differences were considered statistically significant for P-values < 0.05.

## Results and discussion

### Role of substrate stiffness on cell morphological and molecular features

**Figure 2:**
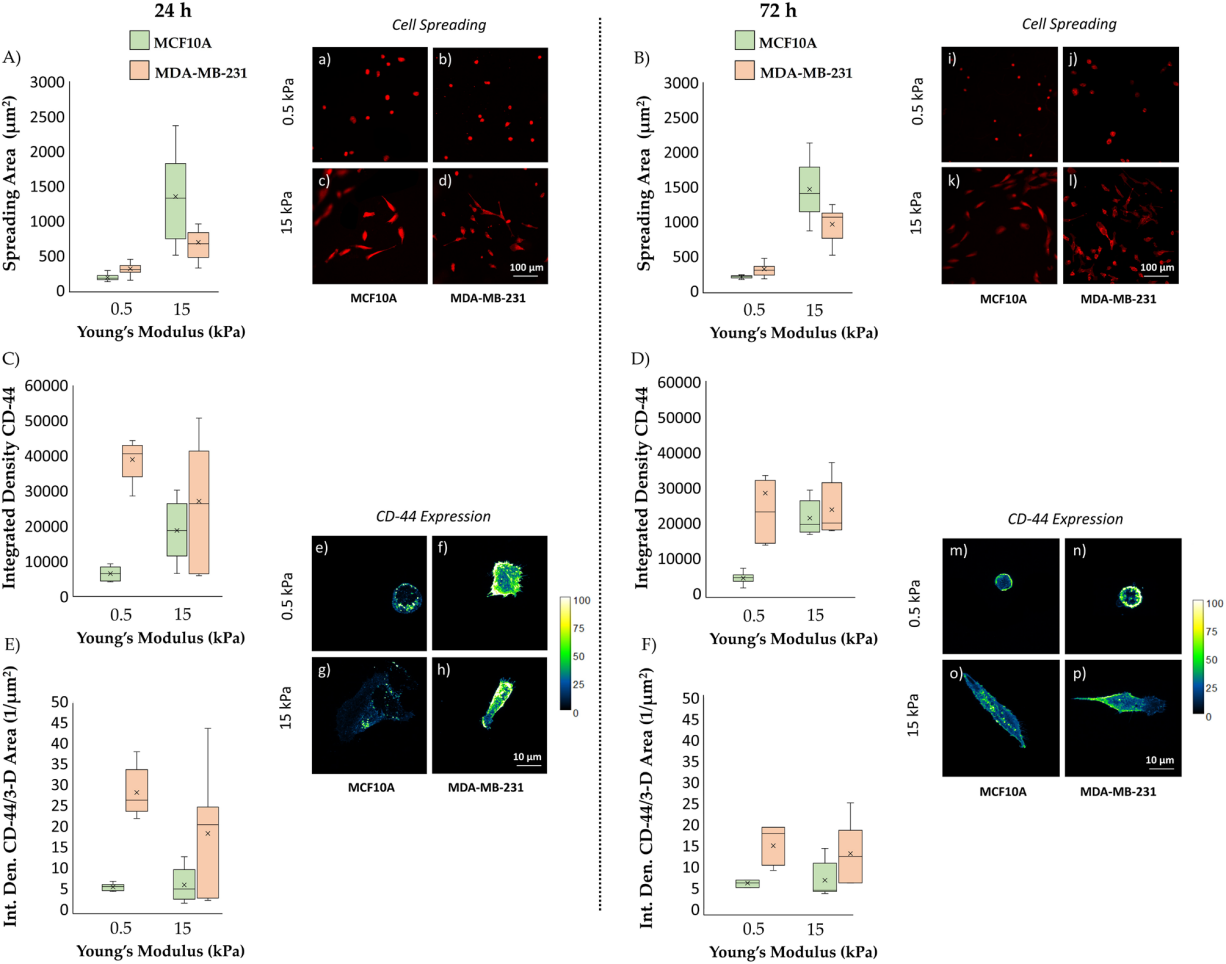
Box plots (mean, median, interquartile range, and outliers) with representative images of spreading areas (**A**,**B**;a–d,i–l) and CD-44 (e–h,m–p) integrated density evaluated both as absolute (**C**,**D**) and normalized by the reconstructed cellular 3D-area (**E**,**F**), for the two cell lines, MCF10A and MDA-MB-231, fixed at 24 h (**A**,**C**,**E**; a–h) and 72 h (**B**,**D**,**F**; i–p) after RT, but not irradiated (control condition). Spreading areas and CD-44 expression were evaluated for two different stiffness of the ECM at 0.5 kPa (a,b,e,f,i,j,m,n) and 15 kPa (c,d,g,h,k,l,o,p). Cells were stained for cellular membrane with WGA-Rhodamine (red) and for CD-44 with Alexa-Fluor 488. The calibration bar fixes the look-up-table (“Green fire Blue”) between 0 (black) and 100 (white) to describe qualitatively the expression of CD-44 for each examined condition. Cells were acquired with a ×10 objective (scale bar, 100 µm) for the spreading analysis (n ≥ 25 for condition), while single cells were acquired with a ×63 objective (scale bar, 10 µm) for the CD-44 expression analysis (n ≥ 8 for condition).

The correlation between cell spreading area and the substrate’s mechanical properties is a pivotal factor in cellular interactions with the microenvironment. As cells adhere to these substrates, they not only establish physical connections, but they also engage in a dynamic dialogue that spans molecular, cellular, and tissue levels. This phenomenon, known as mechano-transduction, allows cells to sense and respond to the mechanical cues presented by their microenvironment. This correlation holds crucial significance for understanding fundamental cellular processes and disease mechanisms. The spreading data obtained for the two cell lines in control conditions (Fig. 2A,B,a–d,i–l; Table S1) reveal their sensing ability in distinguishing between two substrate stiffness levels at both 24 and 72 h. This mechanosensing capability is evident as both cell lines exhibit a noteworthy increase (P < 0.005, as indicated in Table S1) in their adhesion area when cultured on substrates with a stiffness of 15 kPa (Fig. 2A,B,c,d,k,l; Table S1) compared to those with a stiffness of 0.5 kPa (Fig. 2A,B,a,b,i,j; Table S1). Furthermore, we see the evidence of a significant reduction on the adhesion area values for the tumour line compared to those obtained for the healthy line (Fig. 2A,B,a–d,i–l; Table S1). The reduced adhesion of tumour cells, seen especially on the rigid substrate at 15 kPa (Fig. 2A,B,d,l; Table S1), would seem, in fact, to be the cause of the greater cellular deformability which would result in the propensity of these cells to migrate and easily reach even sites distant from the origin site through the processes of invasion and formation of metastases^25,59–62^. In addition, another intriguing phenomenon emerging from this analysis is the relative increase in terms of spreading area which is much more pronounced for the healthy cell line than for the tumour cell line that could be due to a compromised mechanosensing mechanism. This hypothesis is further bolstered by experiments previously conducted by our research group, where we observed the tumour cell line's inability to distinguish between substrates with stiffness levels of 1.3 kPa and 13 kPa^25^. This may indicate the presence of a rigidity threshold that this cell line is capable of sensing^63^. In this context, CD-44 has emerged as a pivotal player in the complex landscape of cancer biology and its expression appears to vary across different conditions. There are variations both within and between cell lines and substrate stiffness levels (Fig. 2C,D,e–p; Table S2). The expression of this protein in the healthy cell line exhibits an upward trend with escalating substrate stiffness (Fig. 2C,D,e,g,m,o; Table S2). This observation aligns with its classification as a membrane protein and correlates with the seen increased spreading area. The tumour line generally shows higher CD-44 expression compared to the healthy counterpart across all conditions (Fig. 2C,D,f,h,n,p; Table S2). The significant difference in CD-44 expression, occurring at both time steps, for the soft substrate between MCF10A and MDA-MB-231 (P < 0.005 as indicated in Table S2) suggests that CD-44 may play a different role in these cell lines' biology, potentially related to their tumour or non-tumour status. Consequently, the upregulation of this protein could serve as a potential target for novel anti-tumour therapies. It is compelling to note that, for the tumour cell line on the substrate simulating the physiological environment, CD-44 expression exhibits a slight decrease over time (Fig. 2C,D,f,n; Table S2). This phenomenon may be attributed to the cell's ongoing reorganization of its shape and alterations in roundness and aspect ratio, both of which are known to play a role in protein expression^64^. Within this framework, and given the substantial heterogeneity observed within tumour populations and tumour microenvironment, it can be useful to decouple the molecular data related to CD-44 expression from the morphological data (Fig. 2E,F; Table S3). The 3D reconstruction of the cell was performed as mentioned before, and the obtained data have been used to normalize the CD-44 expression. The observed trend is in concordance with the preceding data about the integrated density discussed. It reaffirms the consistent overexpression of this protein within the tumour cell line, irrespective of substrate stiffness.

### Effects of RT on spreading area

#### Effects on healthy mammary cells MCF10A

**Figure 3:**
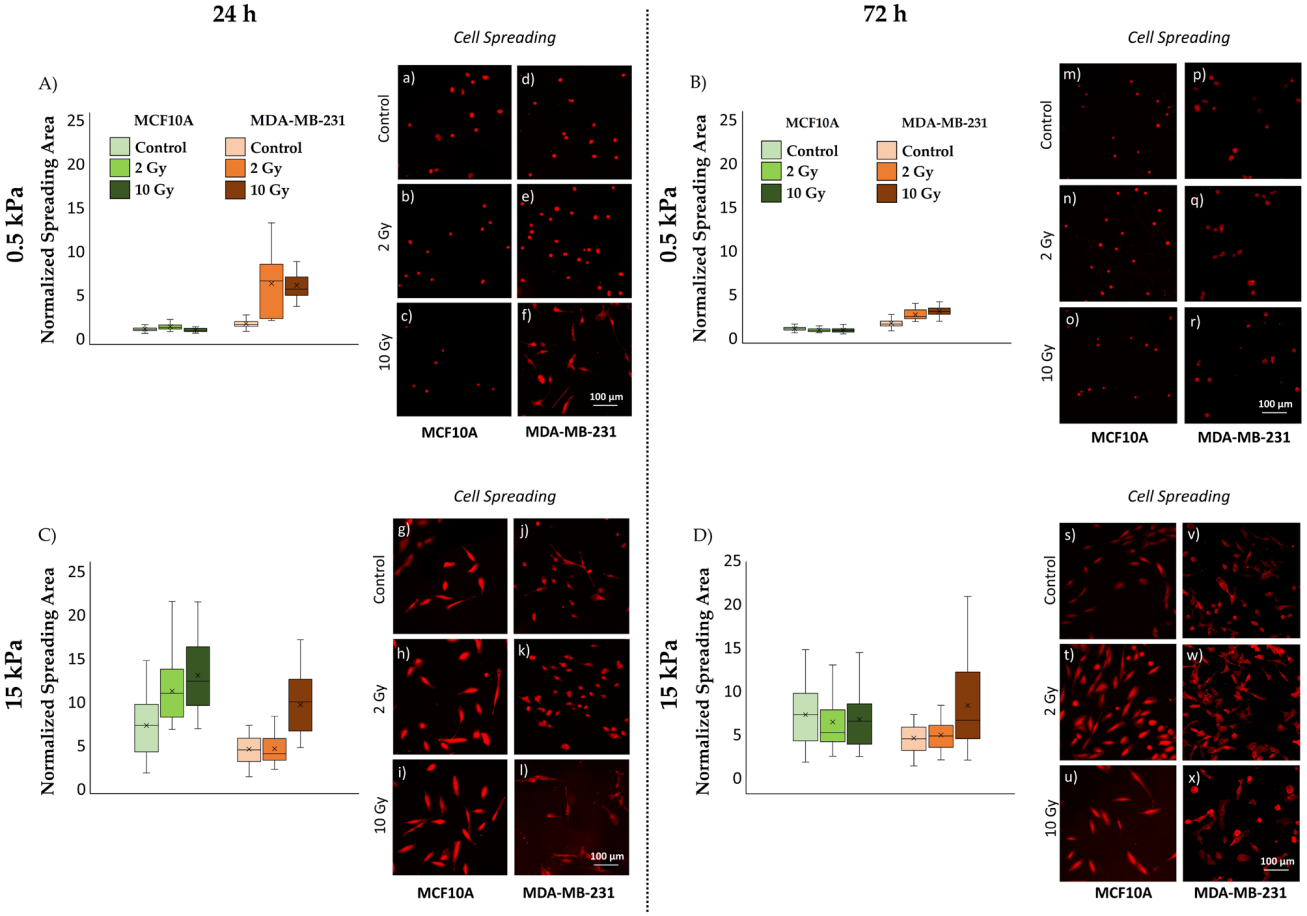
Box plots (mean, median, interquartile range, and outliers) with representative images of spreading areas (**A**–**D**;a–x) for the two cell lines, MCF10A and MDA-MB-231, fixed at 24 h (**A**,**C**; a–l) and 72 h (**B**,**D**; m–x) after RT, evaluated for two different stiffness of the ECM at 0.5 kPa (**A**,**B**; a–f, m–r) and 15 kPa (**C**,**D**; g–l, s–x). Data are represented as normalized by the median of MCF10A cells cultured on 0.5 kPa substrate in control condition. Cells were stained for cellular membrane with WGA-Rhodamine (red) and acquired with a ×10 objective (scale bar, 100 µm). n ≥ 25 for condition.

In our experiments, at 24 h after irradiation, healthy mammary cells (MCF10A) cultured on soft substrates (0.5 kPa) (Fig. 3A,a–c; Table S1) displayed a reduction of the adhesion area linear with the increase of the absorbed dose of radiation, as already proof in our group’s previous work^25^. These results appear to be consistent with a radioprotective role of the soft physiological ECM in case of low-Linear Energy Transfer (low-LET) radiation. In fact, it has been reported that the mechanics of the cellular microenvironment affects the level of cytoskeletal forces on the nucleus. Specifically, the lower ECM stiffness results into a less CSK contractility, leading to a greater degree of DNA compaction^65–67^. From one hand, the presence of more compact DNA domains (i.e., heterochromatin) acts as a physical barrier to reactive radical species produced by water radiolysis responsible for mediating indirect damage to DNA^68^, as reported in previous in vitro studies^69–72^. On the other hand, the augmented degree of DNA compaction restricts the ability of proteins to access and activate DNA repair machinery^73,74^ possibly increasing direct-damage detriment. However, since the percentage of aqueous radiolytic products increase with decreasing LET^75^, we expect a lower contribute of the direct damage component in the case of X-rays irradiation^75–77^. This dual effect requires additional and more specific investigations.

On 15 kPa substrates (Fig. 3C,g–i; Table S1), MCF10A cells exhibited a different response to increasing radiation doses: cytoskeletal tension accumulated in the actomyosin filaments^25,78^ led to an increased cell spreading area in a dose-dependent manner.

At 72 h from irradiation (Fig. 3B,D,m–o,s–u; Table S1), the trends observed at 24 h persisted, with a reduction in the percentage differences in cell spreading between controls and irradiated samples, indicating the repair of a fraction of radiation-induced damage, particularly on rigid substrates. This observation reinforces the role of substrate stiffness and cellular compressibility in the context of radiation response and recovery, especially in the metastatic microenvironment.

#### Effects on tumour cells MDA-MB-231

Tumour cells (MDA-MB-231) displayed a dose-dependent increase in spreading area on both substrate stiffnesses after 24 h from irradiation (Fig. 3A,C,d–f,j–l; Table S1). Unlike healthy cells, the absence of a radioprotective effect in the soft microenvironment of tumour cells suggests alterations in mechanoreceptive properties and intracellular radiation-induced damage repair mechanisms^79,80^.

At 72 h (Fig. 3B,D,p–r,v–x; Table S1), partial recovery of cell spreading was observed in cells irradiated with a low dose of 2 Gy on both stiffness levels. However, when irradiated with a high dose of 10 Gy, cells continued to exhibit significantly greater adhesion areas compared to control conditions.

These findings shed light on the complex interplay between radiation therapy, cell morphology, and the microenvironment, providing valuable insights for future investigations and potential therapeutic strategies.

### Effects of RT on CD-44 expression

The effects of RT on cellular responses are multifaceted and have garnered significant interest. In this section, we delve into the distinct impact of radiation doses and substrate stiffness on both healthy (MCF10A) and tumour (MDA-MB-231) cell lines, shedding light on the intricate interplay between these factors and CD-44 expression dynamics.

#### Effects on healthy cells MCF10A

**Figure 4:**
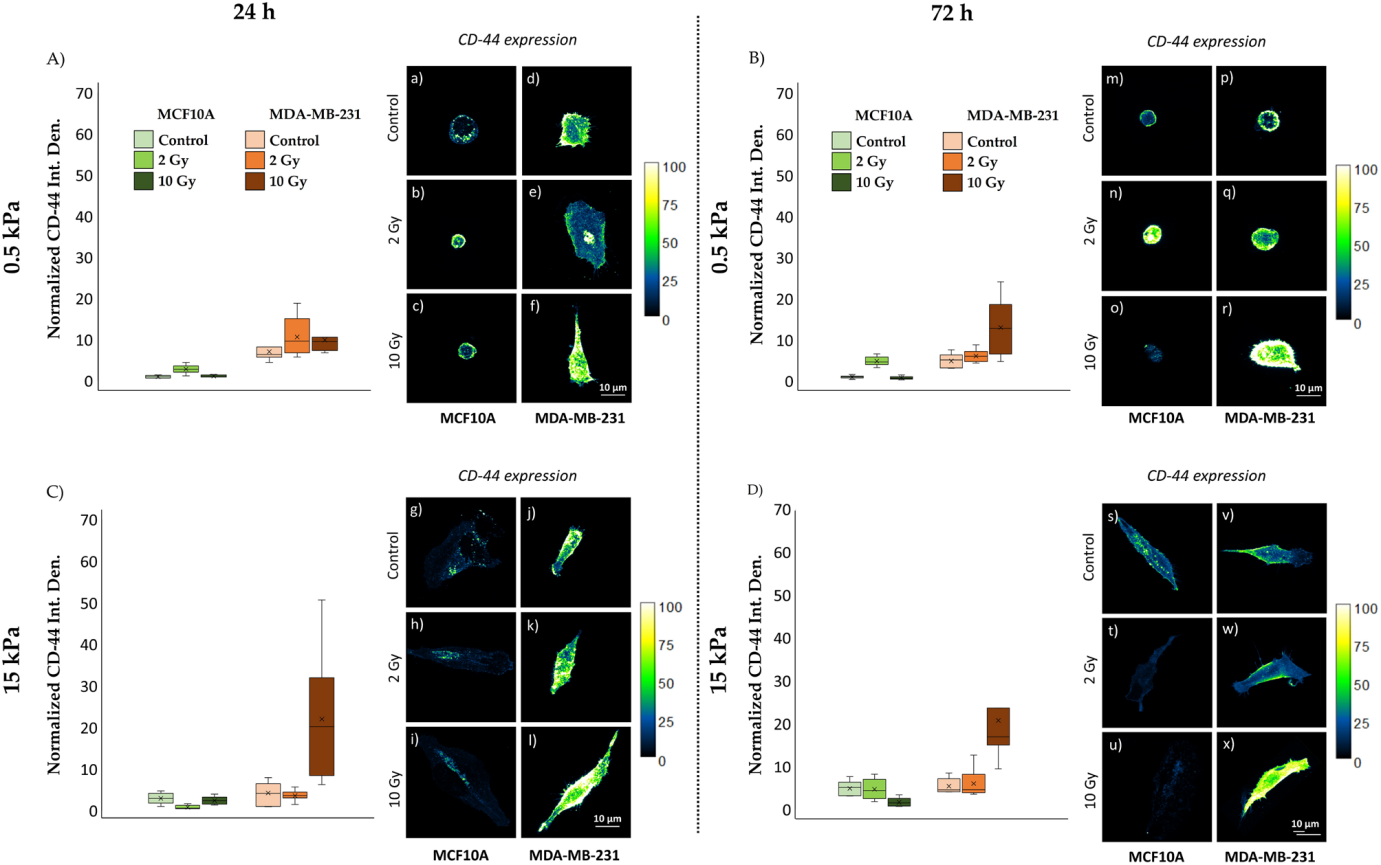
Box plots (mean, median, interquartile range, and outliers) with representative images of CD-44 absolute expression (**A**–**D**; a–x) for the two cell lines, MCF10A and MDA-MB-231, fixed at 24 h (**A**,**C**; a–l) and 72 h (**B**,**D**; m–x) after RT, evaluated for two different stiffness of the ECM at 0.5 kPa (**A**,**B**; a–f, m–r) and 15 kPa (**C**,**D**; g–l, s–x). Data are represented as normalized by the median of MCF10A cells cultured on 0.5 kPa substrate in control condition. Cells were stained for CD-44 with Alexa-Fluor 488 and single cells were acquired with a ×63 objective (scale bar, 10 µm). The calibration bar fixes the look-up-table (“Green fire Blue”) between 0 (black) and 100 (white) to describe qualitatively the expression of CD-44 for each examined condition. n ≥ 8 for condition.

**Figure 5:**
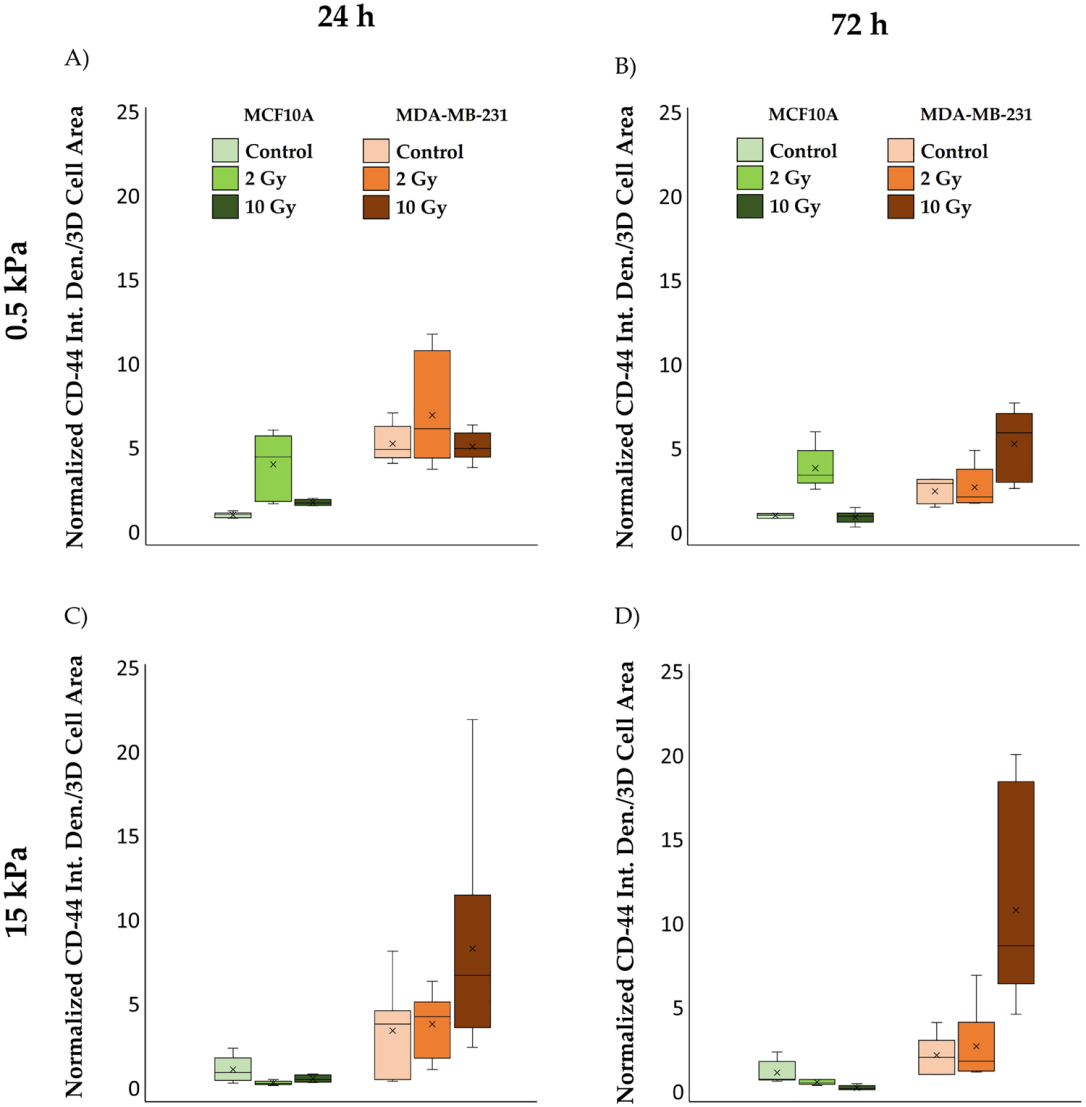
Box plots (mean, median, interquartile range, and outliers) of CD-44 integrated density divided by the reconstructed cellular 3D-area, normalized by the median of MCF10A cells cultured on 0.5 kPa substrate in control condition, for both cell lines, MCF10A and MDA-MB-231, fixed at 24 h (**A**,**C**) and 72 h (**B**,**D**) after irradiation, for two different stiffness of the ECM at 0.5 kPa (**A**,**B**) and 15 kPa (**C**,**D**). n ≥ 8 for condition.

At 24 h post-irradiation (Figs. 4A,a–c, 5A; Tables S2, S3), CD-44 expression in the MCF10A healthy cell line on the soft substrate (0.5 kPa) exhibited a significant increase (P < 0.05, Tables S2, S3) following exposure to an absorbed dose of 2 Gy, compared to the higher dose of 10 Gy. Conversely, on the rigid substrate (15 kPa) (Figs. 4C,g–i, 5C; Tables S2, S3), the normalized CD-44 signal remained constant for the 10 Gy dose, while a significant reduction was observed at 2 Gy (P < 0.05, Tables S2, S3). At 72 h after irradiation (Figs. 4B,m–o, 5B; Tables S2, S3), the trends observed at 24 h remained largely consistent for MCF10A on the soft substrate of 0.5 kPa. However, the decline in CD-44 expression exhibited a linear correlation with the dose, becoming more pronounced on the stiffer substrate at 15 kPa (Figs. 4D,s–u, 5D; Tables S2, S3) The percentage differences in CD-44 relative expression between the two substrates increased with the dose, reaching approximately 94% at 2 Gy and 96% at 10 Gy after 72 h.

#### Effects on tumour cells MDA-MB-231

For the tumour cell line MDA-MB-231, at 24 h post-irradiation, CD-44 expression data did not display significant differences between the three radiation doses, regardless of substrate stiffness (Figs. 4A,C;d–f,j–l, 5A,C; Tables S2, S3). However, after 72 h of RT, a significant increase in CD-44 expression was observed at the higher dose of 10 Gy (Figs. 4B,D; p–r,v–x, 5B,D; Tables S2, S3), regardless of substrate stiffness. The percentage difference in CD-44 expression between the two substrates increased, reaching approximately 98% after 72 h at 10 Gy, emphasizing the substrate's impact on CD-44 expression.

These findings highlight the dynamic changes in CD-44 expression, with percentage differences becoming more pronounced with increasing radiation doses, especially on the stiffer substrate, underscoring the importance of considering these factors in potential therapeutic approaches targeting CD-44.

## Conclusions

In this study, the overexpression of CD-44 was found in breast tumour cells, confirming its potential significance in cancer biology. For the first time, it was reported that RT can exert a direct and discernible influence on CD-44 expression, with a dose-dependent increase observed specifically in the tumour cell line, as opposed to the healthy line. This could help to understand the intricate interplay between RT, CD-44 expression, and cellular spreading behaviour, shedding light on the dynamic response of tumour cells to treatment. Furthermore, the influence of substrate stiffness on CD-44 expression was investigated, by culturing both healthy and tumour cells on stiff substrate mimicking the tumour microenvironment. Our results revealed that the most substantial increase in CD-44 expression occurs precisely in the tumour cell line when exposed to its rigidified microenvironment, coupled with an increase in cellular spreading area.

These findings not only offer insights into elevated radiation resistance and the potential for predicting breast cancer recurrence post-RT but also illuminate a compelling path for future research and clinical utilization. An intriguing avenue could involve investigating CD-44 as a promising therapeutic target, especially in conjunction with an unconventional treatment sequence: radiation therapy followed by chemotherapy. In this regard, the exploration of hyaluronic acid-based nanoparticles with the ability to selectively bind to CD-44 and release chemotherapeutic agents emerges as an encouraging strategy. This innovative approach holds substantial promise for augmenting the effectiveness of cancer treatment protocols, providing renewed optimism in the ongoing battle against cancer.

### Supplementary Information

Supplementary Information.

## Data Availability

The data presented in this study are available on request from the corresponding author.
