# Supplementary material for: Role of the mechanical microenvironment on CD-44 expression of breast adenocarcinoma in response to radiotherapy
Source: Sci Rep. 2024 Jan 3;14:391. doi: 10.1038/s41598-023-50473-x (PMC10764959; doi:10.1038/s41598-023-50473-x)
Supplement: Supplementary file 1 — Supplementary Information. [file 41598_2023_50473_MOESM1_ESM.docx]

Supplementary Materials

Role of the mechanical microenvironment on CD-44 expression of breast adenocarcinoma in response to radiotherapy

Crescenzo Frascogna ^1,2#^, Rocco Mottareale ^3,#^, Giuseppe La Verde ^3,4^, Cecilia Arrichiello ^5^ , Paolo Muto ^5^ , Paolo A. Netti ^1,2,6^, Mariagabriella Pugliese ^3,4,*,§^ Valeria Panzetta ^1,2,6,§^

^1^ Center for Advanced Biomaterials for Healthcare @CRIB, Italian Institute of Technology, Largo Barsanti e Matteucci 53, 80125 Naples, Italy.

^2^ Department of Chemical, Materials and Production Engineering, University of Naples Federico II, Piazzale Vincenzo Tecchio, 80125 Naples, Italy

^3^ Department of Physics “E. Pancini”, University of Naples Federico II, Via Cinthia, 80126 Naples, Italy.

^4^ Istituto Nazionale di Fisica Nucleare, INFN sezione di Napoli, Via Cinthia ed. 6, 80126 Napoli, Italy

^5^ Radiotherapy Unit, Istituto Nazionale Tumouri-IRCCS-Fondazione “G. Pascale”, Via Semmola, 53, 80131 Naples, Italy.

^6^ Interdisciplinary Research Centre on Biomaterials CRIB, University of Naples Federico II, Piazzale Vincenzo Tecchio, 80125 Naples, Italy.

# These authors contributed equally: Crescenzo Frascogna, Rocco Mottareale.

* Author to whom correspondence should be addressed: Mariagabriella Pugliese, mpuglies@na.infn.it

§ These authors share last authorship: Mariagabriella Pugliese, Valeria Panzetta.

**Statistical Analysis**

*Differences between populations of data were evaluated as statistically significant for P-values < 0.05 (highlighted in red)

| **P-values Kruskal-Wallis Test** | | | | **24h** | | | | | | | | | | | |
| --- | --- | --- | --- | --- | --- | --- | --- | --- | --- | --- | --- | --- | --- | --- | --- |
|  |  |  |  | **MCF10A** | | | | | | **MDA-MB-231** | | | | | |
|  |  |  |  | **Control** | | **2 Gy** | | **10 Gy** | | **Control** | | **2 Gy** | | **10 Gy** | |
|  |  |  |  | **0.5 kPa** | **15 kPa** | **0.5 kPa** | **15 kPa** | **0.5 kPa** | **15 kPa** | **0.5 kPa** | **15 kPa** | **0.5 kPa** | **15 kPa** | **0.5 kPa** | **15 kPa** |
| **24h** | **MCF10A** | **Control** | **0.5 kPa** |  | 1,90E-10 | 0.2581 | 0 | 0.788 | 0 | 0.0425 | 0.000004797 | 1,60E-06 | 5,09E-07 | 2,00E-08 | 1,55E-12 |
|  |  |  | **15 kPa** |  |  | 4,99E-05 | 0.06205 | 3,25E-08 | 0.02152 | 1,80E-04 | 0.009471 | 0.4294 | 0.06523 | 0.694 | 0.2707 |
|  |  | **2 Gy** | **0.5 kPa** |  |  |  | 1,55E-10 | 0.2192 | 1,33E-12 | 0.5003 | 0.002898 | 0.00001027 | 0.00002827 | 7,73E-04 | 6,25E-07 |
|  |  |  | **15 kPa** |  |  |  |  | 0 | 0.7081 | 2.38e-13 | 0.000005649 | 0.01012 | 0.0000756 | 0.02638 | 0.5383 |
|  |  | **10 Gy** | **0.5 kPa** |  |  |  |  |  | 0 | 0.04678 | 0.00002587 | 2,79E-05 | 3,94E-05 | 1,02E-06 | 3,10E-10 |
|  |  |  | **15 kPa** |  |  |  |  |  |  | 1.11e-15 | 3.84e-7 | 0.002708 | 0.000005546 | 0.007998 | 0.3304 |
|  | **MDA-MB-231** | **Control** | **0.5 kPa** |  |  |  |  |  |  |  | 0.01126 | 0.00004297 | 0.000118 | 0.000003102 | 2,06E-06 |
|  |  |  | **15 kPa** |  |  |  |  |  |  |  |  | 0.09677 | 0.3186 | 0.03291 | 0.0004138 |
|  |  | **2 Gy** | **0.5 kPa** |  |  |  |  |  |  |  |  |  | 0.3848 | 0.69 | 0.07278 |
|  |  |  | **15 kPa** |  |  |  |  |  |  |  |  |  |  | 0.1742 | 0.003784 |
|  |  | **10 Gy** | **0.5 kPa** |  |  |  |  |  |  |  |  |  |  |  | 0.148 |
|  |  |  | **15 kPa** |  |  |  |  |  |  |  |  |  |  |  |  |
| **72h** | **MCF10A** | **Control** | **0.5 kPa** |  |  |  |  |  |  |  |  |  |  |  |  |
|  |  |  | **15 kPa** |  |  |  |  |  |  |  |  |  |  |  |  |
|  |  | **2 Gy** | **0.5 kPa** |  |  |  |  |  |  |  |  |  |  |  |  |
|  |  |  | **15 kPa** |  |  |  |  |  |  |  |  |  |  |  |  |
|  |  | **10 Gy** | **0.5 kPa** |  |  |  |  |  |  |  |  |  |  |  |  |
|  |  |  | **15 kPa** |  |  |  |  |  |  |  |  |  |  |  |  |
|  | **MDA-MB-231** | **Control** | **0.5 kPa** |  |  |  |  |  |  |  |  |  |  |  |  |
|  |  |  | **15 kPa** |  |  |  |  |  |  |  |  |  |  |  |  |
|  |  | **2 Gy** | **0.5 kPa** |  |  |  |  |  |  |  |  |  |  |  |  |
|  |  |  | **15 kPa** |  |  |  |  |  |  |  |  |  |  |  |  |
|  |  | **10 Gy** | **0.5 kPa** |  |  |  |  |  |  |  |  |  |  |  |  |
|  |  |  | **15 kPa** |  |  |  |  |  |  |  |  |  |  |  |  |
|  |  |  |  |  |  |  |  |  |  |  |  |  |  |  |  |
|  |  |  |  |  |  |  |  |  |  |  |  |  |  |  |  |
| **P-values Kruskal-Wallis Test** | | | | **72h** | | | | | | | | | | | |
|  |  |  |  | **MCF10A** | | | | | | **MDA-MB-231** | | | | | |
|  |  |  |  | **Control** | | **2 Gy** | | **10 Gy** | | **Control** | | **2 Gy** | | **10 Gy** | |
|  |  |  |  | **0.5 kPa** | **15 kPa** | **0.5 kPa** | **15 kPa** | **0.5 kPa** | **15 kPa** | **0.5 kPa** | **15 kPa** | **0.5 kPa** | **15 kPa** | **0.5 kPa** | **15 kPa** |
| **24h** | **MCF10A** | **Control** | **0.5 kPa** | 0.6775 | 1,26E-09 | 0.5411 | 9.77e-15 | 0.5766 | 7.55e-15 | 0.06871 | 1.73e-8 | 0.000736 | 3,51E-07 | 0.0001934 | 3,55E-12 |
|  |  |  | **15 kPa** | 5,99E-06 | 0.5717 | 1,55E-12 | 0.4559 | 1,16E-11 | 0.6774 | 0.000001391 | 0.2997 | 0.0004552 | 0.1496 | 0.003786 | 0.9631 |
|  |  | **2 Gy** | **0.5 kPa** | 0.5564 | 4,12E-05 | 0.09661 | 6,62E-05 | 0.1116 | 3,03E-05 | 0.5412 | 0.00004301 | 0.0491 | 0.00001356 | 0.01868 | 8,70E-06 |
|  |  |  | **15 kPa** | 1,82E-11 | 0.2722 | 0 | 0.003862 | 0 | 0.01315 | 1.54e-11 | 0.005208 | 5,88E-05 | 0.0005489 | 0.000002386 | 0.05538 |
|  |  | **10 Gy** | **0.5 kPa** | 0.5499 | 5,22E-08 | 0.811 | 1,86E-08 | 0.8361 | 9,23E-09 | 0.06696 | 1,78E-04 | 0.001448 | 2,02E-05 | 0.0004317 | 2,87E-09 |
|  |  |  | **15 kPa** | 2.22e-16 | 0.1457 | 0 | 0.0005675 | 0 | 0.002749 | 2.3e-13 | 0.001285 | 2,32E-06 | 0.00006706 | 1.85e-7 | 0.01737 |
|  | **MDA-MB-231** | **Control** | **0.5 kPa** | 0.2031 | 1,61E-04 | 0.00857 | 1,99E-04 | 0.01189 | 9,20E-05 | 0.9888 | 0.0001789 | 0.1481 | 0.00005628 | 0.06064 | 2,66E-05 |
|  |  |  | **15 kPa** | 0.0005529 | 0.003708 | 2,39E-04 | 0.02876 | 6,03E-04 | 0.01522 | 0.01896 | 0.1701 | 0.327 | 0.1968 | 0.6416 | 0.005544 |
|  |  | **2 Gy** | **0.5 kPa** | 0.000001497 | 0.2077 | 4,34E-08 | 0.8544 | 1,51E-07 | 0.641 | 0.0001353 | 0.8043 | 0.01084 | 0.5978 | 0.04471 | 0.3849 |
|  |  |  | **15 kPa** | 0.000003739 | 0.02519 | 4.93e-12 | 0.1927 | 3,31E-08 | 0.1105 | 0.000463 | 0.5681 | 0.04174 | 0.7128 | 0.1538 | 0.0446 |
|  |  | **10 Gy** | **0.5 kPa** | 1,01E-04 | 0.3626 | 3,04E-10 | 0.7787 | 1,51E-09 | 0.9801 | 0.00001485 | 0.5178 | 0.002412 | 0.3236 | 0.01396 | 0.6477 |
|  |  |  | **15 kPa** | 7,35E-08 | 0.6388 | 0 | 0.0564 | 0 | 0.1153 | 2,16E-05 | 0.04407 | 0.00001251 | 0.0127 | 0.0001615 | 0.2688 |
| **72h** | **MCF10A** | **Control** | **0.5 kPa** |  | 5.31e-9 | 0.362 | 7,51E-06 | 0.3872 | 3,45E-06 | 0.2404 | 0.000006794 | 0.01317 | 0.000001805 | 0.004609 | 9,98E-07 |
|  |  |  | **15 kPa** |  |  | 2,40E-11 | 0.2013 | 1,03E-10 | 0.3291 | 8,65E-04 | 0.1389 | 0.0001961 | 0.06081 | 0.001504 | 0.5845 |
|  |  | **2 Gy** | **0.5 kPa** |  |  |  | 0 | 0.975 | 0 | 0.01839 | 6,44E-07 | 0.00008215 | 4,32E-09 | 0.00002011 | 0 |
|  |  |  | **15 kPa** |  |  |  |  | 4,44E-13 | 0.7214 | 0.000002618 | 0.6396 | 0.001296 | 0.3927 | 0.01135 | 0.3969 |
|  |  | **10 Gy** | **0.5 kPa** |  |  |  |  |  | 4,44E-13 | 0.02326 | 1,88E-06 | 0.0001423 | 2,47E-08 | 0.00003581 | 2.22e-16 |
|  |  |  | **15 kPa** |  |  |  |  |  |  | 0.000001186 | 0.4603 | 0.0006231 | 0.2493 | 0.005963 | 0.6225 |
|  | **MDA-MB-231** | **Control** | **0.5 kPa** |  |  |  |  |  |  |  | 0.0004568 | 0.1772 | 0.0002234 | 0.07889 | 3,41E-04 |
|  |  |  | **15 kPa** |  |  |  |  |  |  |  |  | 0.02434 | 0.8057 | 0.0835 | 0.2608 |
|  |  | **2 Gy** | **0.5 kPa** |  |  |  |  |  |  |  |  |  | 0.02243 | 0.642 | 0.000198 |
|  |  |  | **15 kPa** |  |  |  |  |  |  |  |  |  |  | 0.09157 | 0.1161 |
|  |  | **10 Gy** | **0.5 kPa** |  |  |  |  |  |  |  |  |  |  |  | 0.002156 |
|  |  |  | **15 kPa** |  |  |  |  |  |  |  |  |  |  |  |  |

**Table S1.** P-values evaluated with a Kruskal-Wallis test for the statistical analysis of cell spreading areas of MCF10A and MDA-MB-231, fixed at 24 (top) and 72 h (bottom) after radiotherapy cControl, 2 and 10 Gy) for two different substrate stiffnesses of 0.5 and 15 kPa; n ≥ 25.

| **P-values Kruskal-Wallis Test** | | | | **24h** | | | | | | | | | | | |
| --- | --- | --- | --- | --- | --- | --- | --- | --- | --- | --- | --- | --- | --- | --- | --- |
|  |  |  |  | **MCF10A** | | | | | | **MDA-MB-231** | | | | | |
|  |  |  |  | **Control** | | **2 Gy** | | **10 Gy** | | **Control** | | **2 Gy** | | **10 Gy** | |
|  |  |  |  | **0.5 kPa** | **15 kPa** | **0.5 kPa** | **15 kPa** | **0.5 kPa** | **15 kPa** | **0.5 kPa** | **15 kPa** | **0.5 kPa** | **15 kPa** | **0.5 kPa** | **15 kPa** |
| **24h** | **MCF10A** | **Control** | **0.5 kPa** |  | 0.129 | 0.1375 | 0.716 | 0.895 | 0.274 | 0.001561 | 0.03396 | 0.00003651 | 0.03704 | 0.00003051 | 0.00001447 |
|  |  |  | **15 kPa** |  |  | 0.9737 | 0.0701 | 0.1657 | 0.7236 | 0.08623 | 0.5857 | 0.01068 | 0.6102 | 0.009483 | 0.004825 |
|  |  | **2 Gy** | **0.5 kPa** |  |  |  | 0.07511 | 0.176 | 0.7473 | 0.08062 | 0.5624 | 0.009673 | 0.5864 | 0.008579 | 0.004351 |
|  |  |  | **15 kPa** |  |  |  |  | 0.6244 | 0.1629 | 0.0007335 | 0.0168 | 0.00001717 | 0.0184 | 0.00001438 | 0.00000684 |
|  |  | **10 Gy** | **0.5 kPa** |  |  |  |  |  | 0.3331 | 0.002387 | 0.04731 | 0.00006565 | 0.05138 | 0.00005516 | 0.00002615 |
|  |  |  | **15 kPa** |  |  |  |  |  |  | 0.04757 | 0.3769 | 0.005246 | 0.3952 | 0.004646 | 0.002358 |
|  | **MDA-MB-231** | **Control** | **0.5 kPa** |  |  |  |  |  |  |  | 0.209 | 0.5147 | 0.1971 | 0.4898 | 0.3312 |
|  |  |  | **15 kPa** |  |  |  |  |  |  |  |  | 0.03662 | 0.9708 | 0.03294 | 0.01732 |
|  |  | **2 Gy** | **0.5 kPa** |  |  |  |  |  |  |  |  |  | 0.03345 | 0.9659 | 0.71 |
|  |  |  | **15 kPa** |  |  |  |  |  |  |  |  |  |  | 0.03005 | 0.01573 |
|  |  | **10 Gy** | **0.5 kPa** |  |  |  |  |  |  |  |  |  |  |  | 0.7408 |
|  |  |  | **15 kPa** |  |  |  |  |  |  |  |  |  |  |  |  |
| **72h** | **MCF10A** | **Control** | **0.5 kPa** |  |  |  |  |  |  |  |  |  |  |  |  |
|  |  |  | **15 kPa** |  |  |  |  |  |  |  |  |  |  |  |  |
|  |  | **2 Gy** | **0.5 kPa** |  |  |  |  |  |  |  |  |  |  |  |  |
|  |  |  | **15 kPa** |  |  |  |  |  |  |  |  |  |  |  |  |
|  |  | **10 Gy** | **0.5 kPa** |  |  |  |  |  |  |  |  |  |  |  |  |
|  |  |  | **15 kPa** |  |  |  |  |  |  |  |  |  |  |  |  |
|  | **MDA-MB-231** | **Control** | **0.5 kPa** |  |  |  |  |  |  |  |  |  |  |  |  |
|  |  |  | **15 kPa** |  |  |  |  |  |  |  |  |  |  |  |  |
|  |  | **2 Gy** | **0.5 kPa** |  |  |  |  |  |  |  |  |  |  |  |  |
|  |  |  | **15 kPa** |  |  |  |  |  |  |  |  |  |  |  |  |
|  |  | **10 Gy** | **0.5 kPa** |  |  |  |  |  |  |  |  |  |  |  |  |
|  |  |  | **15 kPa** |  |  |  |  |  |  |  |  |  |  |  |  |
|  |  |  |  |  |  |  |  |  |  |  |  |  |  |  |  |
|  |  |  |  |  |  |  |  |  |  |  |  |  |  |  |  |
| **P-values Kruskal-Wallis Test** | | | | **72h** | | | | | | | | | | | |
|  |  |  |  | **MCF10A** | | | | | | **MDA-MB-231** | | | | | |
|  |  |  |  | **Control** | | **2 Gy** | | **10 Gy** | | **Control** | | **2 Gy** | | **10 Gy** | |
|  |  |  |  | **0.5 kPa** | **15 kPa** | **0.5 kPa** | **15 kPa** | **0.5 kPa** | **15 kPa** | **0.5 kPa** | **15 kPa** | **0.5 kPa** | **15 kPa** | **0.5 kPa** | **15 kPa** |
| **24h** | **MCF10A** | **Control** | **0.5 kPa** | 0.6313 | 0.06749 | 0.06363 | 0.04157 | 0.4793 | 0.989 | 0.02405 | 0.04205 | 0.01214 | 0.02676 | 0.0001237 | 0.0006128 |
|  |  |  | **15 kPa** | 0.03548 | 0.7564 | 0.7364 | 0.555 | 0.01979 | 0.1517 | 0.5265 | 0.6067 | 0.351 | 0.3293 | 0.02671 | 0.0714 |
|  |  | **2 Gy** | **0.5 kPa** | 0.03868 | 0.7314 | 0.7117 | 0.5341 | 0.02173 | 0.1609 | 0.5038 | 0.5838 | 0.3336 | 0.3161 | 0.02439 | 0.06603 |
|  |  |  | **15 kPa** | 0.9456 | 0.03511 | 0.03299 | 0.02148 | 0.7766 | 0.7177 | 0.01161 | 0.02133 | 0.005795 | 0.01443 | 0.00005749 | 0.0002814 |
|  |  | **10 Gy** | **0.5 kPa** | 0.5346 | 0.08981 | 0.08493 | 0.05588 | 0.3961 | 0.9108 | 0.03442 | 0.0573 | 0.01774 | 0.03509 | 0.0002177 | 0.001021 |
|  |  |  | **15 kPa** | 0.106 | 0.516 | 0.4999 | 0.3661 | 0.06696 | 0.3011 | 0.3292 | 0.3983 | 0.2106 | 0.2158 | 0.01333 | 0.03719 |
|  | **MDA-MB-231** | **Control** | **0.5 kPa** | 0.0001365 | 0.1557 | 0.1631 | 0.2813 | 0.0000558 | 0.002568 | 0.2216 | 0.2207 | 0.3745 | 0.6327 | 0.782 | 0.909 |
|  |  |  | **15 kPa** | 0.005432 | 0.8234 | 0.8448 | 0.9262 | 0.002547 | 0.04545 | 0.9403 | 0.9914 | 0.6867 | 0.5754 | 0.08431 | 0.1952 |
|  |  | **2 Gy** | **0.5 kPa** | 7,86E-04 | 0.02567 | 0.02755 | 0.06948 | 2,27E-04 | 0.00009292 | 0.0372 | 0.04351 | 0.09167 | 0.2897 | 0.6655 | 0.3881 |
|  |  |  | **15 kPa** | 0.0061 | 0.851 | 0.8725 | 0.8997 | 0.002884 | 0.04918 | 0.9102 | 0.9805 | 0.6599 | 0.5562 | 0.07772 | 0.1824 |
|  |  | **10 Gy** | **0.5 kPa** | 6,26E-04 | 0.02307 | 0.02478 | 0.06369 | 1,79E-04 | 0.000079 | 0.03336 | 0.03941 | 0.08373 | 0.2749 | 0.6337 | 0.3643 |
|  |  |  | **15 kPa** | 3,12E-04 | 0.01213 | 0.01307 | 0.03599 | 9,12E-05 | 0.00003772 | 0.01732 | 0.02124 | 0.04633 | 0.1849 | 0.4252 | 0.2262 |
| **72h** | **MCF10A** | **Control** | **0.5 kPa** |  | 0.01491 | 0.01379 | 0.008816 | 0.8058 | 0.6389 | 0.003123 | 0.007973 | 0.00139 | 0.007011 | 0.000003093 | 0.00002457 |
|  |  |  | **15 kPa** |  |  | 0.9789 | 0.7683 | 0.007767 | 0.08373 | 0.7629 | 0.8379 | 0.5415 | 0.4702 | 0.05956 | 0.1412 |
|  |  | **2 Gy** | **0.5 kPa** |  |  |  | 0.7877 | 0.007139 | 0.07932 | 0.7845 | 0.8586 | 0.5597 | 0.4835 | 0.06348 | 0.149 |
|  |  |  | **15 kPa** |  |  |  |  | 0.004587 | 0.05264 | 0.9784 | 0.9208 | 0.7829 | 0.6491 | 0.141 | 0.2797 |
|  |  | **10 Gy** | **0.5 kPa** |  |  |  |  |  | 0.4934 | 0.001369 | 0.003967 | 0.0005942 | 0.004004 | 9,09E-04 | 0.000008026 |
|  |  |  | **15 kPa** |  |  |  |  |  |  | 0.03377 | 0.05428 | 0.01785 | 0.0329 | 0.0002925 | 0.001235 |
|  | **MDA-MB-231** | **Control** | **0.5 kPa** |  |  |  |  |  |  |  | 0.9339 | 0.7326 | 0.6075 | 0.08741 | 0.2065 |
|  |  |  | **15 kPa** |  |  |  |  |  |  |  |  | 0.6904 | 0.5788 | 0.09585 | 0.2104 |
|  |  | **2 Gy** | **0.5 kPa** |  |  |  |  |  |  |  |  |  | 0.8043 | 0.1903 | 0.3795 |
|  |  |  | **15 kPa** |  |  |  |  |  |  |  |  |  |  | 0.4541 | 0.6749 |
|  |  | **10 Gy** | **0.5 kPa** |  |  |  |  |  |  |  |  |  |  |  | 0.6557 |
|  |  |  | **15 kPa** |  |  |  |  |  |  |  |  |  |  |  |  |

**Table S2.** P-values evaluated with a Kruskal-Wallis test for the statistical analysis of CD-44 expression of MCF10A and MDA-MB-231, fixed at 24 (top) and 72 h (bottom) after radiotherapy (control, 2 and 10 Gy) for two different substrate stiffnesses of 0.5 and 15 kPa; n ≥ 8.

| **P-values Kruskal-Wallis Test** | | | | **24h** | | | | | | | | | | | |
| --- | --- | --- | --- | --- | --- | --- | --- | --- | --- | --- | --- | --- | --- | --- | --- |
|  |  |  |  | **MCF10A** | | | | | | **MDA-MB-231** | | | | | |
|  |  |  |  | **Control** | | **2 Gy** | | **10 Gy** | | **Control** | | **2 Gy** | | **10 Gy** | |
|  |  |  |  | **0.5 kPa** | **15 kPa** | **0.5 kPa** | **15 kPa** | **0.5 kPa** | **15 kPa** | **0.5 kPa** | **15 kPa** | **0.5 kPa** | **15 kPa** | **0.5 kPa** | **15 kPa** |
| **24h** | **MCF10A** | **Control** | **0.5 kPa** |  | 0.9791 | 0.0161 | 0.269 | 0.3484 | 0.4932 | 0.00161 | 0.07981 | 0.0003318 | 0.02178 | 0.001382 | 0.0008057 |
|  |  |  | **15 kPa** |  |  | 0.0173 | 0.2583 | 0.362 | 0.4775 | 0.001761 | 0.08461 | 0.0003682 | 0.02339 | 0.001519 | 0.0008854 |
|  |  | **2 Gy** | **0.5 kPa** |  |  |  | 0.0006739 | 0.1419 | 0.002884 | 0.4547 | 0.4559 | 0.275 | 0.839 | 0.4834 | 0.345 |
|  |  |  | **15 kPa** |  |  |  |  | 0.04556 | 0.6875 | 0.0000391 | 0.004991 | 0.00000528 | 0.0008906 | 0.00002889 | 0.00001706 |
|  |  | **10 Gy** | **0.5 kPa** |  |  |  |  |  | 0.1143 | 0.02666 | 0.4362 | 0.008896 | 0.1865 | 0.02607 | 0.01582 |
|  |  |  | **15 kPa** |  |  |  |  |  |  | 0.000222 | 0.01764 | 0.00003808 | 0.003871 | 0.0001786 | 0.0001044 |
|  | **MDA-MB-231** | **Control** | **0.5 kPa** |  |  |  |  |  |  |  | 0.1282 | 0.752 | 0.3276 | 0.9403 | 0.844 |
|  |  |  | **15 kPa** |  |  |  |  |  |  |  |  | 0.05583 | 0.5723 | 0.1322 | 0.08443 |
|  |  | **2 Gy** | **0.5 kPa** |  |  |  |  |  |  |  |  |  | 0.1777 | 0.6842 | 0.911 |
|  |  |  | **15 kPa** |  |  |  |  |  |  |  |  |  |  | 0.3467 | 0.2368 |
|  |  | **10 Gy** | **0.5 kPa** |  |  |  |  |  |  |  |  |  |  |  | 0.7802 |
|  |  |  | **15 kPa** |  |  |  |  |  |  |  |  |  |  |  |  |
| **72h** | **MCF10A** | **Control** | **0.5 kPa** |  |  |  |  |  |  |  |  |  |  |  |  |
|  |  |  | **15 kPa** |  |  |  |  |  |  |  |  |  |  |  |  |
|  |  | **2 Gy** | **0.5 kPa** |  |  |  |  |  |  |  |  |  |  |  |  |
|  |  |  | **15 kPa** |  |  |  |  |  |  |  |  |  |  |  |  |
|  |  | **10 Gy** | **0.5 kPa** |  |  |  |  |  |  |  |  |  |  |  |  |
|  |  |  | **15 kPa** |  |  |  |  |  |  |  |  |  |  |  |  |
|  | **MDA-MB-231** | **Control** | **0.5 kPa** |  |  |  |  |  |  |  |  |  |  |  |  |
|  |  |  | **15 kPa** |  |  |  |  |  |  |  |  |  |  |  |  |
|  |  | **2 Gy** | **0.5 kPa** |  |  |  |  |  |  |  |  |  |  |  |  |
|  |  |  | **15 kPa** |  |  |  |  |  |  |  |  |  |  |  |  |
|  |  | **10 Gy** | **0.5 kPa** |  |  |  |  |  |  |  |  |  |  |  |  |
|  |  |  | **15 kPa** |  |  |  |  |  |  |  |  |  |  |  |  |
|  |  |  |  |  |  |  |  |  |  |  |  |  |  |  |  |
|  |  |  |  |  |  |  |  |  |  |  |  |  |  |  |  |
| **P-values Kruskal-Wallis Test** | | | | **72h** | | | | | | | | | | | |
|  |  |  |  | **MCF10A** | | | | | | **MDA-MB-231** | | | | | |
|  |  |  |  | **Control** | | **2 Gy** | | **10 Gy** | | **Control** | | **2 Gy** | | **10 Gy** | |
|  |  |  |  | **0.5 kPa** | **15 kPa** | **0.5 kPa** | **15 kPa** | **0.5 kPa** | **15 kPa** | **0.5 kPa** | **15 kPa** | **0.5 kPa** | **15 kPa** | **0.5 kPa** | **15 kPa** |
| **24h** | **MCF10A** | **Control** | **0.5 kPa** | 0.8185 | 0.7982 | 0.009058 | 0.5925 | 0.9385 | 0.25 | 0.08391 | 0.1529 | 0.04398 | 0.0945 | 0.0005037 | 0.00001794 |
|  |  |  | **15 kPa** | 0.8398 | 0.8185 | 0.009777 | 0.5753 | 0.9609 | 0.2398 | 0.0889 | 0.1605 | 0.04692 | 0.09978 | 0.000559 | 0.00002027 |
|  |  | **2 Gy** | **0.5 kPa** | 0.02333 | 0.03149 | 0.8389 | 0.004658 | 0.01257 | 0.0005711 | 0.4419 | 0.3285 | 0.629 | 0.4627 | 0.3649 | 0.07319 |
|  |  |  | **15 kPa** | 0.1735 | 0.1773 | 0.0003259 | 0.5851 | 0.2123 | 0.9656 | 0.005346 | 0.01357 | 0.002236 | 0.00694 | 0.000007833 | 1,81E-04 |
|  |  | **10 Gy** | **0.5 kPa** | 0.4571 | 0.4953 | 0.0945 | 0.1529 | 0.3548 | 0.04091 | 0.45 | 0.6229 | 0.2978 | 0.4627 | 0.01327 | 0.0009133 |
|  |  |  | **15 kPa** | 0.3541 | 0.3528 | 0.001505 | 0.8858 | 0.4232 | 0.6561 | 0.01873 | 0.04054 | 0.008729 | 0.02263 | 0.00005736 | 0.000001719 |
|  | **MDA-MB-231** | **Control** | **0.5 kPa** | 0.002336 | 0.003751 | 0.5863 | 0.0003963 | 0.000985 | 0.00003214 | 0.1224 | 0.0846 | 0.2081 | 0.1383 | 0.9149 | 0.3097 |
|  |  |  | **15 kPa** | 0.1131 | 0.1372 | 0.3388 | 0.02656 | 0.07144 | 0.004315 | 0.9806 | 0.7885 | 0.7847 | 0.9868 | 0.08064 | 0.008269 |
|  |  | **2 Gy** | **0.5 kPa** | 0.0004707 | 0.0008882 | 0.3785 | 0.00007381 | 0.0001583 | 0.000004227 | 0.05279 | 0.03524 | 0.1012 | 0.06376 | 0.8196 | 0.4663 |
|  |  |  | **15 kPa** | 0.03162 | 0.04247 | 0.6788 | 0.00627 | 0.01704 | 0.0007529 | 0.5559 | 0.4175 | 0.7707 | 0.5762 | 0.2445 | 0.03787 |
|  |  | **10 Gy** | **0.5 kPa** | 0.002001 | 0.003358 | 0.6242 | 0.0003277 | 0.0007861 | 0.00002352 | 0.126 | 0.08637 | 0.2178 | 0.1434 | 0.8477 | 0.2563 |
|  |  |  | **15 kPa** | 0.001163 | 0.001967 | 0.4587 | 0.0001914 | 0.0004565 | 0.00001391 | 0.08031 | 0.05469 | 0.1434 | 0.09321 | 0.9176 | 0.4169 |
| **72h** | **MCF10A** | **Control** | **0.5 kPa** |  | 0.9713 | 0.01318 | 0.4404 | 0.868 | 0.1592 | 0.1187 | 0.2098 | 0.06321 | 0.1321 | 0.0007157 | 0.00002385 |
|  |  |  | **15 kPa** |  |  | 0.01857 | 0.436 | 0.8444 | 0.1632 | 0.1434 | 0.2405 | 0.08031 | 0.1567 | 0.001349 | 0.0000573 |
|  |  | **2 Gy** | **0.5 kPa** |  |  |  | 0.002498 | 0.006669 | 0.0002739 | 0.3271 | 0.2379 | 0.4876 | 0.3484 | 0.491 | 0.1139 |
|  |  |  | **15 kPa** |  |  |  |  | 0.5212 | 0.5558 | 0.02811 | 0.05767 | 0.01361 | 0.03321 | 0.0001115 | 0.00000368 |
|  |  | **10 Gy** | **0.5 kPa** |  |  |  |  |  | 0.1953 | 0.07547 | 0.1468 | 0.03709 | 0.08718 | 0.0002386 | 0.000005895 |
|  |  |  | **15 kPa** |  |  |  |  |  |  | 0.004626 | 0.01196 | 0.001912 | 0.006056 | 0.000006257 | 1,40E-04 |
|  | **MDA-MB-231** | **Control** | **0.5 kPa** |  |  |  |  |  |  |  | 0.8065 | 0.7661 | 0.9946 | 0.07638 | 0.007695 |
|  |  |  | **15 kPa** |  |  |  |  |  |  |  |  | 0.5956 | 0.8083 | 0.05111 | 0.005023 |
|  |  | **2 Gy** | **0.5 kPa** |  |  |  |  |  |  |  |  |  | 0.7802 | 0.143 | 0.0179 |
|  |  |  | **15 kPa** |  |  |  |  |  |  |  |  |  |  | 0.0908 | 0.01066 |
|  |  | **10 Gy** | **0.5 kPa** |  |  |  |  |  |  |  |  |  |  |  | 0.3268 |
|  |  |  | **15 kPa** |  |  |  |  |  |  |  |  |  |  |  |  |

**Table S3.** P-values evaluated with a Kruskal-Wallis test for the statistical analysis of CD-44 expression normalized by the reconstructed cellular 3D-area of MCF10A and MDA-MB-231, fixed at 24 (top) and 72 h (bottom) after radiotherapy (control, 2 and 10 Gy) for two different substrate stiffnesses of 0.5 and 15 kPa; n ≥ 8.


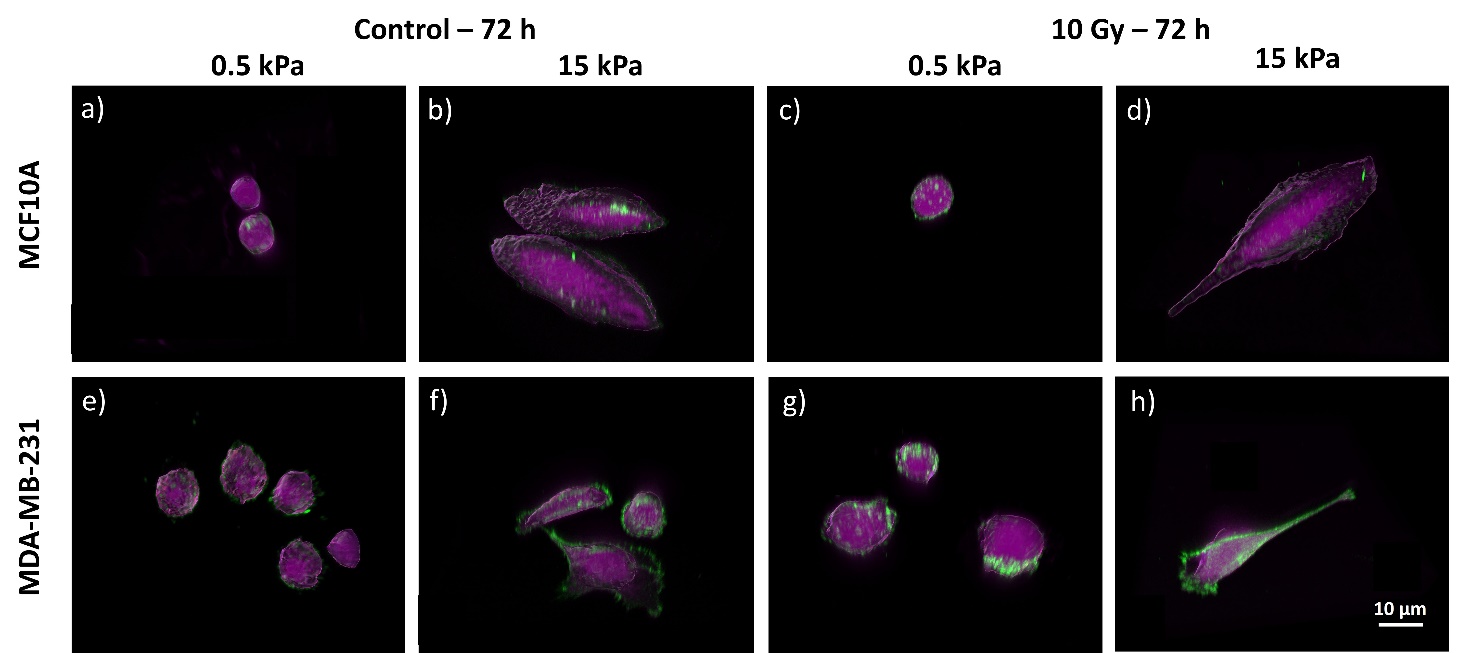


**Figure S1.** *Representative images of 3D cellular reconstructions. Single cells were stained for cellular membrane with Cell Tracker™ Red CMTPX (violet) and for CD-44 with AntiCD-44 (anti-mouse)-Alexa Fluor 488 (green).*
